# Left-sided location is a risk factor for lymph node metastasis of T1 colorectal cancer: a single-center retrospective study

**DOI:** 10.1007/s00384-020-03668-x

**Published:** 2020-06-16

**Authors:** Kenichi Mochizuki, Shin-ei Kudo, Katsuro Ichimasa, Yuta Kouyama, Shingo Matsudaira, Yuki Takashina, Yasuharu Maeda, Tomoyuki Ishigaki, Hiroki Nakamura, Naoya Toyoshima, Yuichi Mori, Masashi Misawa, Noriyuki Ogata, Toyoki Kudo, Takemasa Hayashi, Kunihiko Wakamura, Naruhiko Sawada, Fumio Ishida, Hideyuki Miyachi

**Affiliations:** Digestive Disease Center, Showa University Northern Yokohama Hospital, 35-1 Chigasaki-chuo, Tsuzuki, Yokohama, 224-8503 Japan

**Keywords:** T1 colorectal cancer, Lymph node metastasis, Left-sided colon, Risk factor

## Abstract

**Purpose:**

Although some studies have reported differences in clinicopathological features between left- and right-sided advanced colorectal cancer (CRC), there are few reports regarding early-stage disease. In this study, we aimed to compare the clinicopathological features of left- and right-sided T1 CRC.

**Methods:**

Subjects were 1142 cases with T1 CRC undergoing surgical or endoscopic resection between 2001 and 2018 at Showa University Northern Yokohama Hospital. Of these, 776 cases were left-sided (descending colon to rectum) and 366 cases were right-sided (cecum to transverse colon). We compared clinical (patients age, sex, tumor size, morphology, initial treatment) and pathological features (invasion depth, histological grade, lymphatic invasion, vascular invasion, tumor budding) including lymph node metastasis (LNM).

**Results:**

Left-sided T1 CRC showed significantly higher rates of LNM (left-sided 12.0% vs. right-sided 5.4%, *P* < 0.05) and lymphatic invasion (left-sided 32.7% vs. right-sided 23.2%, *P* < 0.05). Especially, the sigmoid colon and rectum showed higher rates of LNM (12.4% and 12.1%, respectively) than other locations. Patients with left-sided T1 CRC were younger than those with right-sided T1 CRC (64.9 years ±11.5 years vs. 68.7 ± 11.6 years, *P* < 0.05), as well as significantly lower rates of poorly differentiated carcinoma/mucinous carcinoma than right-sided T1 CRC (11.6% vs. 16.1%, *P* < 0.05).

**Conclusion:**

Left-sided T1 CRC, especially in the sigmoid colon and rectum, exhibited higher rates of LNM than right-sided T1 CRC, followed by higher rates of lymphatic invasion. These results suggest that tumor location should be considered in decisions regarding additional surgery after endoscopic resection.

**Trial registration:**

This study was registered with the University Hospital Medical Network Clinical Trials Registry (UMIN 000032733).

## Introduction

Colorectal cancer (CRC) is one of the most common cancers worldwide. In Japan, the incidence and mortality of CRC have increased during the last several decades. CRC is the most common cause of cancer death in female and the third most common in male [1]. Moreover, CRC mortality is the fourth highest in male and the third highest in female globally, and the second highest worldwide when considering male and female in total [2]. The differentiation of CRC by anatomical location has received substantial attention. CRC is divided into two sides: left-sided, which is composed of the descending and sigmoid colon and the rectum, and right-sided, containing the cecum and the ascending and transverse colon. Many publications have highlighted differences between left- and right-sided CRC [3–7].

Compared with right-sided CRC, left-sided advanced (T2–T4) CRC has the following features: longer overall survival; younger patients; higher number of male patients; less mucinous or poorly differentiated histology; less associated with *BRAF* and *APC* mutations, microsatellite instability (MSI), and CpG island methylator phenotype (CIMP); and better response to anti-epidermal growth factor receptor (EGFR) therapies. In addition, apart from having a different embryological origin, the proximal colon from the midgut and the distal colon and the rectum from the hindgut, the left-sided colon displays peculiar differences in its mucosal immunology, probably because of differences in the gut microbiota [8–13].

Many studies have investigated the differences in epidemiology, pathogenesis, genetic alterations, and molecular pathways between left- and right-sided advanced CRC, as well as those that divided the location of CRC into the colon and rectum [14–16]. However, few articles have focused on the differences in early-(T1) stage disease and divided the tumor location into the left- and right-sided colon. Therefore, the present study was designed to evaluate the clinicopathological characteristics between left- and right-sided T1 CRC.

## Materials and methods

### Patients

**Fig. 1:**
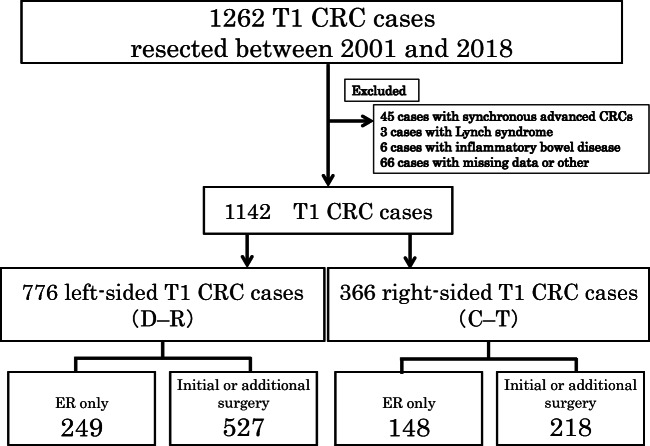
Study flow chart. CRC, colorectal cancer; ER, endoscopic resection

Between April 2001 and December 2018, a total of 1262 T1 CRC cases were resected endoscopically or surgically at Showa University Northern Yokohama Hospital, Japan. Written informed consent was obtained from all the patients prior to endoscopy. Our ethics committee approved the study protocol (approval number: 17H107). This study was registered with the University Hospital Medical Network Clinical Trials Registry (UMIN 000032733). Patients who underwent surgery because of a synchronous invasive carcinoma (*n* = 45) and those who were diagnosed with Lynch syndrome (*n* = 3) or ulcerative colitis (*n* = 6) were excluded. In some cases, detailed pathological evaluation was not possible owing to the loss of or damage to the specimen, and thus these were also excluded. Furthermore, we did not include patients who received preoperative chemotherapy or radiotherapy. In total, 1142 cases were included. Of these, 776 cases were left-sided (descending colon to rectum), and 366 cases were right-sided (cecum to transverse colon). We analyzed clinicopathological features including patient age, sex, tumor size, morphology, initial treatment, depth of invasion, histological grade, vascular invasion, lymphatic invasion, tumor budding, and lymph node metastasis (LNM). We classified tumor morphology into three types according to the Paris classification and Kudo’s classification: flat type (IIa, laterally spreading tumor), protruded type (Is, Ip, Isp), and depressed type (IIc, IIa+IIc, IIc+IIa, Is+IIc, Ip+IIc). [17] Surgical specimens were used as the standard for the presence of LNM. For patients with endoscopic resection, local lymph node recurrent cases detected by CT or MRI were defined as LNM-positive, regardless of the period. In the endoscopic resection alone group, the mean follow-up period was 41.5 ± 34.7 months (Fig. 1).

### Histological examination

All resected specimens were retrieved and immediately fixed in a 10% buffered formalin and were observed with a focus on the pit pattern using a stereomicroscope. They were then cut at the point where the deepest invasion area could be exposed on the cut end surface. The other histological specimens were cut into parallel 2- to 3-mm-thick sections and stained with hematoxylin and eosin (H&E). Tumor size was measured after formalin fixation. All specimens were diagnosed based on the World Health Organization Classification of Tumors [18] and the current JSCCR (Japanese Society for Cancer of the Colon and Rectum) guidelines [19]. Histological grade was classified in view of the World Health Organization criteria as follows: well-differentiated adenocarcinoma, moderately differentiated adenocarcinoma, poorly differentiated adenocarcinoma (Por), and mucinous carcinoma (Muc). In this study, a Por/Muc component was considered to be present if any part of the lesion contained any of these features. Vascular invasion was diagnosed by double staining with H&E and Victoria blue (Muto Pure Chemicals Co., Ltd., Tokyo, Japan) and lymphatic invasion was diagnosed by H&E staining and immunostaining with D2-40 antibody (Dako North America Inc., Carpinteria, CA, USA). Tumor budding was defined as a cancer cell nest consisting of one to five cells at the invasive margin of the carcinoma. After selecting the field where budding was the most intensive, the number of buddings was counted with a × 20 objective lens. Depending on the number of buddings, budding grade was scored as follows: BD1, 0–4; BD2, 5–9; and BD3, ≥ 10. BD 2–3 was defined as tumor budding-positive [20, 21]. The depth of submucosal invasion was assessed according to the JSCCR classification as < 1000 μm (T1a) and ≥ 1000 μm (T1b) [19].

### Statistical analysis

Nominal and ordinal variables are expressed as frequencies and percentages. Continuous variables are reported as mean ± standard deviation (SD). Continuous variables were compared using Student’s *t* tests, whereas dichotomous variables were compared using chi-squared or Fisher exact tests, as appropriate. Multivariate logistic regression analysis regarding LNM was subsequently performed to calculate odds ratios and 95% confidence intervals (CIs). All statistical analyses were performed using JMP Pro version 14.0.0 (SAS Institute Inc., Cary, NC, USA). All *P* values were two sided, and *P* < 0.05 was considered statistically significant.

## Results

### Clinicopathological features of left- and right-sided T1 CRC

The clinicopathological characteristics of 1142 patients are shown in Table 1. Table 2 shows the clinicopathological features of left- and right-sided T1 CRC. Left-sided T1 CRC showed significantly higher rates of LNM (left-sided 12.0% vs. right-sided 5.4%, *P* < 0.05) and was accompanied by higher rates of lymphatic invasion (left-sided 32.7% vs. right-sided 23.2%, *P* < 0.05) than right-sided T1 CRC. Left-sided T1 CRC also showed higher rates of T1b cases (left-sided 78.4% vs. right-sided 68.3%, *P* < 0.05) and surgical resections (left-sided 67.9% vs. right-sided 59.6%, *P* < 0.05). In morphology, left-sided T1 CRC showed significantly lower rates of flat type morphology (left-sided 27.6% vs. right-sided 53.0%, *P* < 0.05). In contrast, the protruded type was significantly more frequent in left-sided T1 CRC (left-sided 51.2% vs. right-sided 20.5%, *P* < 0.05). For pathological features, left-sided T1 CRC showed significantly lower rates of Por/Muc (left-sided 11.6% vs. right-sided 16.1%, *P* < 0.05). Patients with left-sided T1 CRC were younger (left-sided 64.9 ± 11.5 years vs. right-sided 68.7 ± 11.6 years, *P* < 0.05) than those with right-sided T1 CRC. No significant differences were found in the other factors.

**Table 1:** Clinicopathological characteristics of the study patients (*n* = 1142)

Age (years)	66.1 ± 11.6
Sex (male)	723 (63.3)
Location (left-sided)	776 (68.0)
Tumor size (mm)	21.5 ± 13.6
Morphology (flat type/protruded/depressed)	408 (35.7)/472 (41.3)/262 (22.9)
Initial treatment (endoscopic resection)	735 (64.4)
Treatment (surgical resection^a^)	745 (65.2)
Depth of invasion (T1b)	858 (75.1)
Histological grade (Poror Muc^b^)	149 (13.0)
Vascular invasion (+)	305 (26.7)
Lymphatic invasion (+)	339 (29.7)
Tumor budding (BD 2 or 3)	236 (20.7)
Lymph node metastasis (+)^c^	75 (10.1)

Results are expressed as mean ± standard deviation or number of patients (%), as appropriate

^a^Surgical resection: initial and additional surgical resection

^b^Por or Muc, poorly differentiated adenocarcinoma or mucinous carcinoma

^c^Includes only surgical cases. The denominator is the number of each surgery

**Table 2:** Clinicopathological characteristics of patients with left- and right-sided T1 CRC

	Left (*n* = 776)	Right (*n* = 366)	*P* value
Age (years)	64.9 ± 11.5	68.7 ± 11.6	< 0.05
Sex (male)	491 (63.3)	232 (63.4)	0.97
Tumor size (mm)	21.0 ± 14.2	22.5 ± 12.6	0.08
Morphology (flat type/protruded/depressed)	214 (27.6)/397 (51.2)/165 (21.3)	194 (53.0)/75 (20.5)/97 (26.5)	< 0.05
Initial treatment (endoscopic resection)	510 (65.7)	225 (61.5)	0.16
Treatment (surgical resection^a^)	527 (67.9)	218 (59.6)	< 0.05
Depth of invasion (T1b)	608 (78.4)	250 (68.3)	< 0.05
Histological grade (Por or Muc^b^)	90 (11.6)	59 (16.1)	< 0.05
Vascular invasion (+)	216 (27.8)	89 (24.3)	0.21
Lymphatic invasion (+)	254 (32.7)	85 (23.2)	< 0.05
Tumor budding (BD 2 or 3)	171 (22.3)	65 (17.8)	0.10
Lymph node metastasis (+)^c^ (T1a/T1b)	63 (12.0) (5/58)	12 (5.4) (0/12)	< 0.05
Local lymph node recurrence (+)^d^ (T1a/T1b)	5 (2.0) (1/4)	0 (0) (0/0)	0.162

Results are expressed as mean ± standard deviation or number of patients (%), as appropriate

^a^Surgical resection: initial and additional surgical resection

^b^Por or Muc, poorly differentiated adenocarcinoma or mucinous carcinoma

^c^Includes only surgical cases. The denominator is the number of each surgery

^d^Includes only endoscopic resection cases. The denominator is the number of each endoscopic resection

Table 3 shows the clinicopathological features of left- and right-sided T1 CRC without the JSCCR guidelines’ curative cases, which had one or more risk factors such as lymphovascular invasion, tumor budding, Por/Muc component, or T1b. Higher rates of initial endoscopic resection, lymphatic invasion, and LNM were evident in left-sided T1 CRC.

**Table 3:** Clinicopathological characteristics of patients with left- and right-sided T1 CRC without the Japanese guidelines’ curative cases

	Left (*n* = 652)	Right (*n* = 278)	*P* value
Age (years)	64.9 ± 11.5	68.6 ± 11.8	< 0.05
Sex (male)	409 (62.7)	166 (59.7)	0.39
Tumor size (mm)	21.6 ± 14.7	22.2 ± 12.3	0.59
Morphology (flat type/protruded/depressed)	167 (25.6)/329 (50.5)/156 (23.9)	127 (45.7)/62 (22.3)/89 (32.0)	< 0.05
Initial treatment (endoscopic resection)	399 (61.2)	147 (52.9)	< 0.05
Treatment (surgical resection^a^)	509 (78.1)	206 (74.1)	0.19
Depth of invasion (T1b)	606 (92.9)	250 (89.9)	0.12
Histological grade (Por or Muc^b^)	89 (13.7)	58 (20.9)	< 0.05
Vascular invasion (+)	216 (33.1)	89 (32.0)	0.74
Lymphatic invasion (+)	254 (38.9)	85 (30.6)	< 0.05
Tumor budding (BD 2 or 3)	171 (26.2)	65 (23.4)	0.36
Lymph node metastasis (+)^c^	61 (9.4)	12 (4.3)	< 0.05
Local lymph node recurrence (+)^d^ (T1a/T1b)	0 (0) (0/0)	0 (0) (0/0)	N/A

Results are expressed as mean ± standard deviation or number of patients (%), as appropriate

*N/A*, not applicable

^a^Surgical resection: initial and additional surgical resection

^b^Por or Muc, poorly differentiated adenocarcinoma or mucinous carcinoma

^c^Includes only surgical cases. The denominator is the number of each surgery

^d^Includes only endoscopic resection cases. The denominator is the number of each endoscopic resection

We also compared the differences in clinicopathological features by each location in Table 4. The sigmoid colon and rectum showed higher positive rates of lymphatic invasion and tumor budding than other sites. The rectum also showed the highest rate of vascular invasion (38.9%). Patients’ age was younger in sigmoid (64.9 ± 11.2) and rectum (63.9 ± 11.5) T1 CRC. Fewer cases were operated upon in the descending colon (46.9%), whereas more cases were operated upon in the sigmoid and rectum (70.3% and 66.8%, respectively). T1 CRC of the sigmoid colon and rectum showed a higher rate of LNM (12.4% and 12.1%) than other sites. The frequency of LNM with each morphology at both locations is shown in Table 5. Sigmoid lesions of the depressed type showed the highest percentage of LNM (13.2%).

**Table 4:** Clinicopathological characteristics of T1 CRC according to each location

	C (*n* = 61)	A (*n* = 168)	T (*n* = 137)	D (*n* = 49)	S (*n* = 516)	R (*n* = 211)
Age (years)	68.8 ± 11.2	68.7 ± 11.5	68.6 ± 11.9	68.6 ± 12.9	64.9 ± 11.2	63.9 ± 11.5
Sex (male)	32 (52.5)	108 (64.3)	92 (67.2)	35 (71.4)	322 (62.4)	134 (63.5)
Tumor size (mm)	26.3 ± 11.4	22.8 ± 14.0	20.6 ± 11.0	20.0 ± 9.8	19.0 ± 10.8	26.2 ± 19.8
Morphology						
Flat type	32 (52.5)	88 (52.4)	74 (54.0)	26 (53.1)	108 (20.9)	80 (37.9)
Protruded type	17 (27.9)	34 (20.2)	24 (17.5)	18 (36.7)	302 (58.5)	77 (36.5)
Depressed type	12 (19.7)	46 (27.4)	39 (28.5)	5 (10.2)	106 (20.5)	54 (25.6)
Initial treatment (endoscopic resection)	38 (62.3)	101 (60.1)	86 (62.8)	34 (69.4)	352 (68.2)	124 (58.8)
Treatment (surgical resection^a^)	38 (62.3)	104 (61.9)	76 (55.5)	23 (46.9)	363 (70.3)	141 (66.8)
Depth of invasion (T1b)	43 (70.5)	119 (70.8)	88 (64.2)	29 (59.2)	401 (77.7)	178 (84.4)
Histological grade (Por or Muc^b^)	12 (19.7)	22 (13.1)	25 (18.2)	2 (4.1)	63 (12.2)	25 (11.8)
Vascular invasion (+)	13 (21.3)	43 (25.6)	33 (24.1)	5 (10.2)	129 (25.0)	82 (38.9)
Lymphatic invasion (+)	14 (23.0)	38 (22.6)	33 (24.1)	8 (16.3)	169 (32.8)	77 (36.5)
Tumor budding (BD 2 or 3)	10 (16.4)	32 (19.0)	23 (16.8)	7 (14.3)	114 (22.1)	50 (23.7)
Lymph node metastasis (+)^c^	2/38 (5.3)	4/104 (3.8)	6/76 (7.9)	1/23 (4.3)	45/363 (12.4)	17/141 (12.1)
Local lymph node recurrence (+)^d^	0/23 (0)	0/64 (0)	0/61 (0)	0/26 (0)	1/153 (0.7)	4/70 (5.7)

Results are expressed as mean ± standard deviation or number of patients (%), as appropriate

*C*, cecum; *A*, ascending colon; *T*, transverse colon, *D*, descending colon; *S*, sigmoid colon; *R*, rectum

^a^Surgical resection: initial and additional surgical resection

^b^Por or Muc, poorly differentiated adenocarcinoma or mucinous carcinoma

^c^Includes only surgical cases. The denominator is the number of each surgery

^d^Includes only endoscopic resection cases. The denominator is the number of each endoscopic resection

**Table 5:** LNM or LNR according to each location

	C (*n* = 61)	A (*n* = 168)	T (*n* = 137)	D (*n* = 49)	S (*n* = 516)	R (*n* = 211)
Flat type						
LNM (+)^a^	1/32 (3.1)	1/88 (1.1)	1/74 (1.4)	0/26 (0)	6/108 (5.6)	5/80 (6.3)
LNR (+)^b^	0/15 (0)	0/45 (0)	0/46 (0)	0/14 (0)	0/43 (0)	2/40 (5.0)
Protruded type						
LNM (+)^a^	1/17 (5.9)	2/34 (5.9)	2/24 (8.3)	1/18 (5.6)	25/302 (8.3)	8/77 (10.4)
LNR (+)^b^	0/7 (0)	0/13 (0)	0/9 (0)	0/11 (0)	1/99 (1.0)	0/23 (0)
Depressed type						
LNM (+)^a^	0/12 (0)	1/46 (2.2)	3/39 (7.7)	0/5 (0)	14/106 (13.2)	4/54 (7.4)
LNR (+)^b^	0/1 (0)	0/6 (0)	0/6 (0)	0/1 (0)	0/11 (0)	2/7 (28.6)

Results are expressed as number of patients (%)

*C*, cecum; *A*, ascending colon; *T*, transverse colon; *D*, descending colon; *S*, sigmoid colon; *R*, rectum; *LNM*, lymph node metastasis; *LNR*, local lymph node recurrence

^a^Includes only surgical cases. The denominator is the number of each surgery

^b^Includes only endoscopic resection cases. The denominator is the number of each endoscopic resection

Table 6 shows the relationships between clinicopathological factors and LNM identified by univariate and multivariate logistic analyses. Left-sided location was an independent risk factor for LNM (OR, 2.42; 95% CI, 1.23–4.78). Furthermore, lymphovascular invasion, histological grade, and sex were independent risk factors for LNM in T1 CRC.

**Table 6:** Relationships between clinicopathological factors and LNM

	Univariate analysis			Multivariate analysis	
	LNM+ (*n* = 75)	LNM− (*n* = 670)	*P* value	OR (95% CI)	*P* value
Age (< 70)	47 (62.7)	413(61.6)	0.86	0.84 (0.50–1.43)	0.53
Sex (male)	38 (81.3)	422 (63.0)	< 0.05	0.57 (0.34–0.97)	< 0.05
Tumor size (≥ 20 mm)	40 (53.3)	326 (48.7)	0.44	1.25 (0.71–2.20)	0.43
Location (left)	63 (84.0)	464 (69.3)	< 0.05	2.42 (1.23–4.78)	< 0.05
Morphology (depressed type)	22 (29.3)	208 (31.0)	0.76	0.78 (0.39–1.57)	0.49
Initial treatment (endoscopic resection)	37 (49.3)	304 (45.4)	0.51	0.94 (0.51–1.71)	0.83
Depth of invasion (T1b)	70 (93.3)	619 (92.4)	0.77	0.74 (0.26–2.12)	0.57
Histological grade (Por or Muc^a^)	21 (28.0)	102 (15.2)	< 0.05	2.08 (1.13–3.81)	< 0.05
Lymphovascular invasion (+)	71 (94.7)	350 (52.2)	< 0.05	15.5 (5.47–44.1)	< 0.05
Tumor budding (BD 2 or 3)	38 (50.7)	168 (25.1)	< 0.05	1.64 (0.97–2.79)	0.07

Results are expressed as number of patients (%)

*LNM*, lymph node metastasis; *OR*, odds ratio; *CI*, confidence interval

^a^Por or Muc, poorly differentiated adenocarcinoma or mucinous carcinoma

**Fig. 2:**
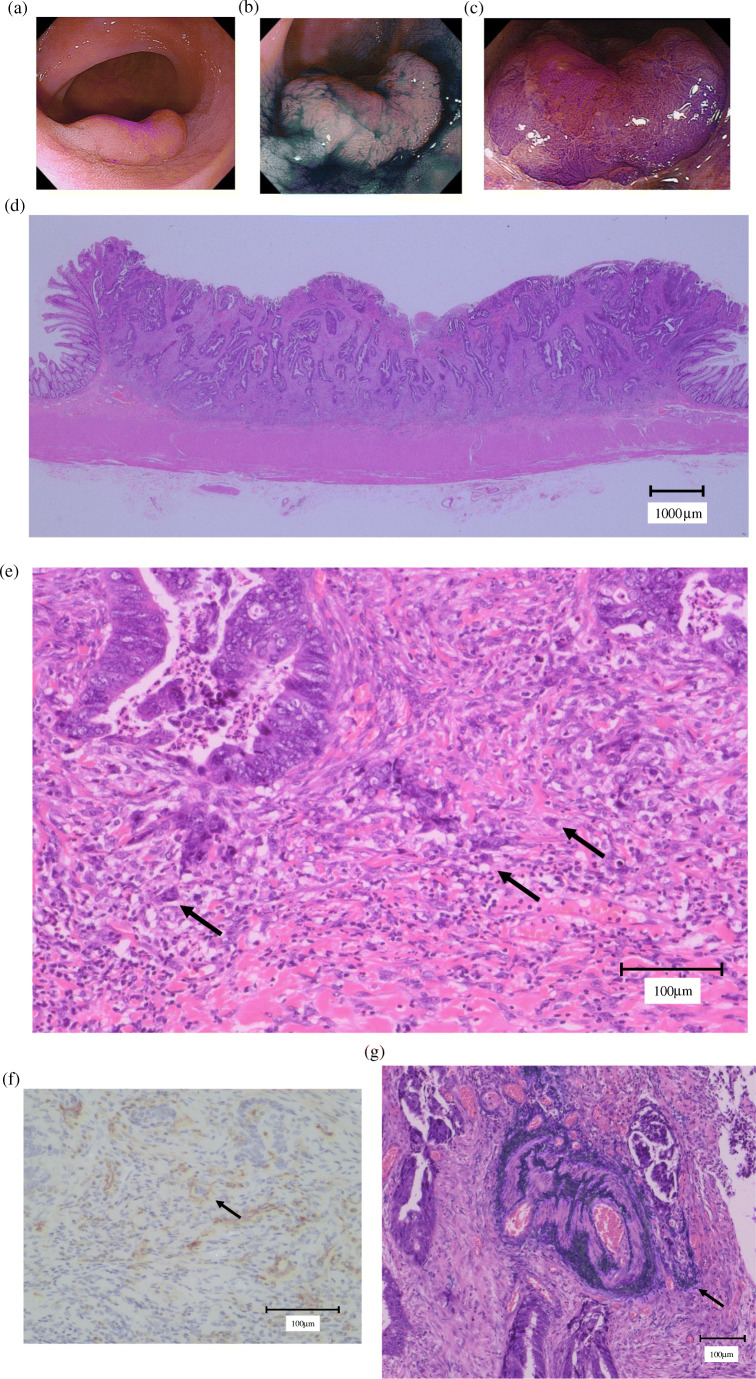
A typical case of left-sided T1 CRC. **a** A 15-mm-sized lesion of erythematous color was viewed by white light observation. This lesion was located in the sigmoid colon. **b** During indigo carmine spray observation, the dye accumulated in the circumferential grooved margin. The lesion was diagnosed as IIa+IIc due to the margin of depression. **c** In magnified observation with crystal violet staining, non-structure area was found around irregular pits and it was diagnosed as *V*_N_ type pit pattern. Initial laparoscopic-assisted surgery was performed. **d** Histology of this lesion by HE staining. The infiltrative advanced region had a fused tubular structure, and the worst histological diagnosis was moderately differentiated adenocarcinoma. **e** Tumor budding was evident in HE staining. **f** Histology of the lesion by D2-40 staining. Lymphatic invasion was evident. **g** Histology of the lesion by Victoria Blue staining. Vascular invasion was apparent. **h** Histology of metastatic lymph nodes by HE staining. The pathological diagnosis was T1 carcinoma (SM 2375 μm), type IIa+IIc, 19 mm, papillary and well-to-moderately differentiated adenocarcinoma, ly1, v1, BD2, pN1

Figure 2 shows images of a typical left-sided T1 CRC case, demonstrating its lymphovascular invasion-positive, tumor budding-positive, and LNM-positive features.

## Discussion

In this study, we compared the clinicopathological features of left-sided T1 CRC with right-sided CRC. Left-sided T1 CRC had a significantly higher rate of lymphatic invasion and LNM. More careful management would be required for left-sided T1 CRC when determining the need for additional surgery after endoscopic resection.

We focused on T1 CRC and revealed that the left-sided location was a risk factor for LNM in T1 CRC. One reason that the clinicopathological features differed by location may be anatomical and genetic differences. The gastrointestinal tract is derived from the endoderm, from which the left colon is derived from the hindgut, while the right colon is derived from the midgut. In advanced cancer, some studies reported the following differences between left- and right-sided CRC. Left-sided CRC patients have a better PFS (progression-free survival), OS (overall survival), and ORR (overall response rate) than right-sided CRC patients. As a histological grade, there are lower rates of mucinous carcinomas, and these express a serrated pathway signature in left-sided CRC. Left-sided CRC was present in a lower percentage of female and had multiple metastatic sites. However, most of these studies investigated advanced CRC, and there is no obvious mechanism regarding these differences in early-stage disease. Similar to advanced CRC, younger age and a lower rate of Por/Muc were observed in left-sided T1 CRC. In this study, the positive rate of lymphatic invasion was high in the left side, suggesting that this may be a factor in the high rate of LNM in left-sided T1 CRC.

There have been various studies regarding the risk of LNM [22–26]. Lymphovascular invasion and histological grade are described in guidelines from the USA, Europe, and Japan [19, 27–30]. Furthermore, in Europe and Japan, the degree of SM invasion and in Japan, budding grade are described as risk factors. In addition, female sex and the status of the muscularis mucosae were also reported as risk factors [22, 31–33]. Regarding location, there have been several reports comparing the rectum and colon, which showed that the rate of LNM was equivalent or higher in the rectum [15, 16]. The current study dividing the tumor location into the left- and right-side is the largest study of T1 CRC. As a result, the left-sided location was an independent risk factor for LNM. Especially, the positive rate of LNM was highest in the sigmoid colon (12.4%) and rectum (12.1%), whereas the descending colon showed 4.3% LNM. Loree et al. reported characteristics that differed by location among left-sided sites. For example, many descending colon features appear more “right-sided.” There was a higher proportion of mucinous histology in the descending colon (24%) compared with the sigmoid (14%) and rectosigmoid junction (12%) [34]. These features suggest that while the descending colon is classified on the left, it may be closer to the right-sided colon. In any case, the results of our study suggested that tumor location should be considered when determining the need for additional surgery after endoscopic resection of T1 CRC.

The present study had several limitations. First, this was a retrospective analysis based on clinical records. Regarding LNM in patients treated by endoscopic resection alone, we used the findings of CT or MRI instead. However, our cohort was one of the largest single-center studies of T1 CRC. Second, the statistical power might be insufficient to reveal small differences in each tumor location subgroup analysis. Third, the pathological diagnosis of the included patients was not re-evaluated. A large-scale multicenter study is needed to verify the clinicopathological features of left- and right-sided CRC revealed in this study.

In conclusion, this study indicated that left-sided T1 CRC, especially that in the sigmoid colon and rectum, shows higher rates of LNM than right-sided T1 CRC, followed by higher rates of lymphatic invasion. These results suggested that left-sided T1 CRC should be considered different diseases that require different forms of management.
